# OP05 Societal Preferences For Prioritizing Patients Suffering From Breast Cancer, Deafness, Or Knee Arthrosis For Scarce Surgical Capacity

**DOI:** 10.1017/S0266462325100640

**Published:** 2025-12-29

**Authors:** Vivian Reckers-droog, Anouk van Alphen, Saskia Knies, Robert Baatenburg de Jong, Thomas Reindersma

## Abstract

**Introduction:**

Constraints on surgical capacity due to budgetary and workforce shortages necessitate prioritization. Lessons learned from the COVID-19 pandemic emphasize the societal debate around these decisions and stress the need to align decisions with societal preferences. This study examined societal preferences for prioritizing patients with three different conditions—breast cancer, deafness, or knee arthrosis—for scarce surgical capacity.

**Methods:**

We conducted a labeled discrete choice experiment among 1,046 members of the Dutch public. Respondents completed 14 choice tasks in which they prioritized patients for surgery, based on condition, age, health-related quality of life (HRQoL) before and after surgery, and waiting time until surgery.

**Results:**

Respondents were more likely to prioritize patients suffering from breast cancer over patients suffering from knee arthrosis or deafness. Respondents were also more likely to prioritize patients with lower levels of HRQoL before surgery, larger surgery-related increases in HRQoL, and longer waiting times until surgery. They were less likely to prioritize patients who were relatively older, although the opposite held true for patients suffering from deafness. Observed preference heterogeneity largely resulted from differences in preference strength, rather than preference direction.

**Conclusions:**

Our results provided insight into societal preferences for prioritizing patients with different conditions for surgery. This insight aids in understanding public outcry that may follow decisions deviating from societal preferences. Aligning prioritization decisions with societal preferences may increase their legitimacy. Further research may examine the relevance of these preferences for physicians and their willingness to be guided by this.

